# Supplementary material for: Comparing the Influence of Assembly Processes Governing Bacterial Community Succession Based on DNA and RNA Data
Source: Microorganisms. 2020 May 26;8(6):798. doi: 10.3390/microorganisms8060798 (PMC7355735; doi:10.3390/microorganisms8060798)
Supplement: Supplementary file 1 [file microorganisms-08-00798-s001.pdf]

microorganisms

Appendix A. Supplementary data

The following is the supplementary data related to the article: *Comparing the influence of assembly processes governing bacterial community succession based on DNA and RNA data*, authored by Xiu Jia, Francisco Dini-Andreote and Joana Falcão Salles

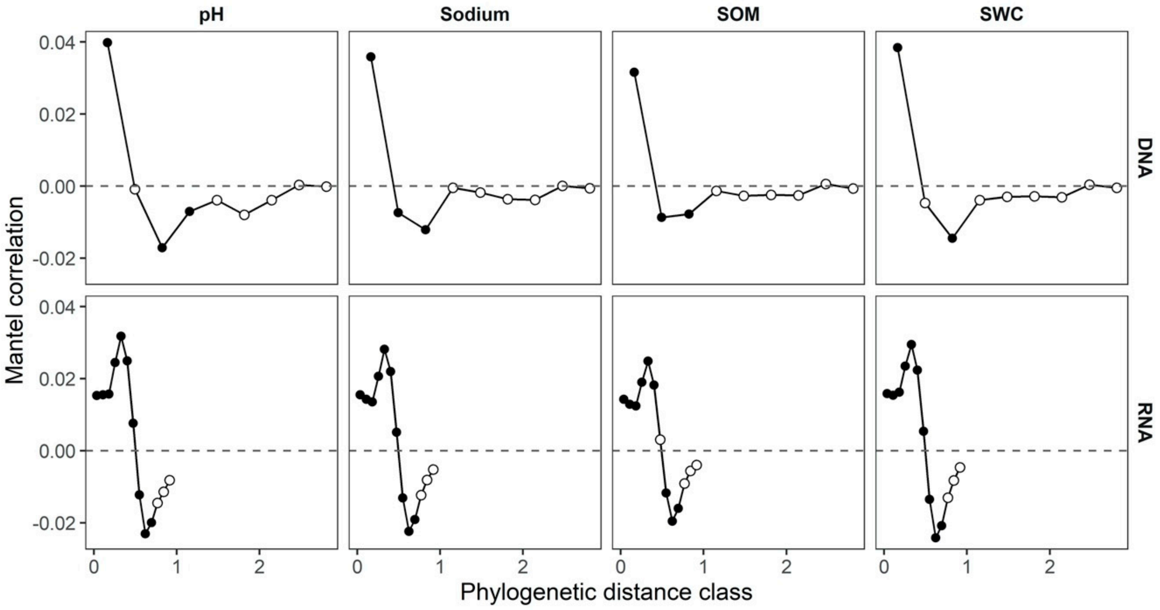

Figure S1 Mantel correlogram showing the correlation between phylogenetic distances Amplicon Sequence Variants (ASVs) and the environmental optima of ASVs, which was based on pH, sodium concentration (Sodium), soil organic matter (SOM) and soil water content (SWC). Correlograms were presented for the DNA (upper panel) and RNA (lower panel) datasets, respectively. Solid symbols indicate significant correlations ($P < 0.01$), and open circles indicate non-significant.

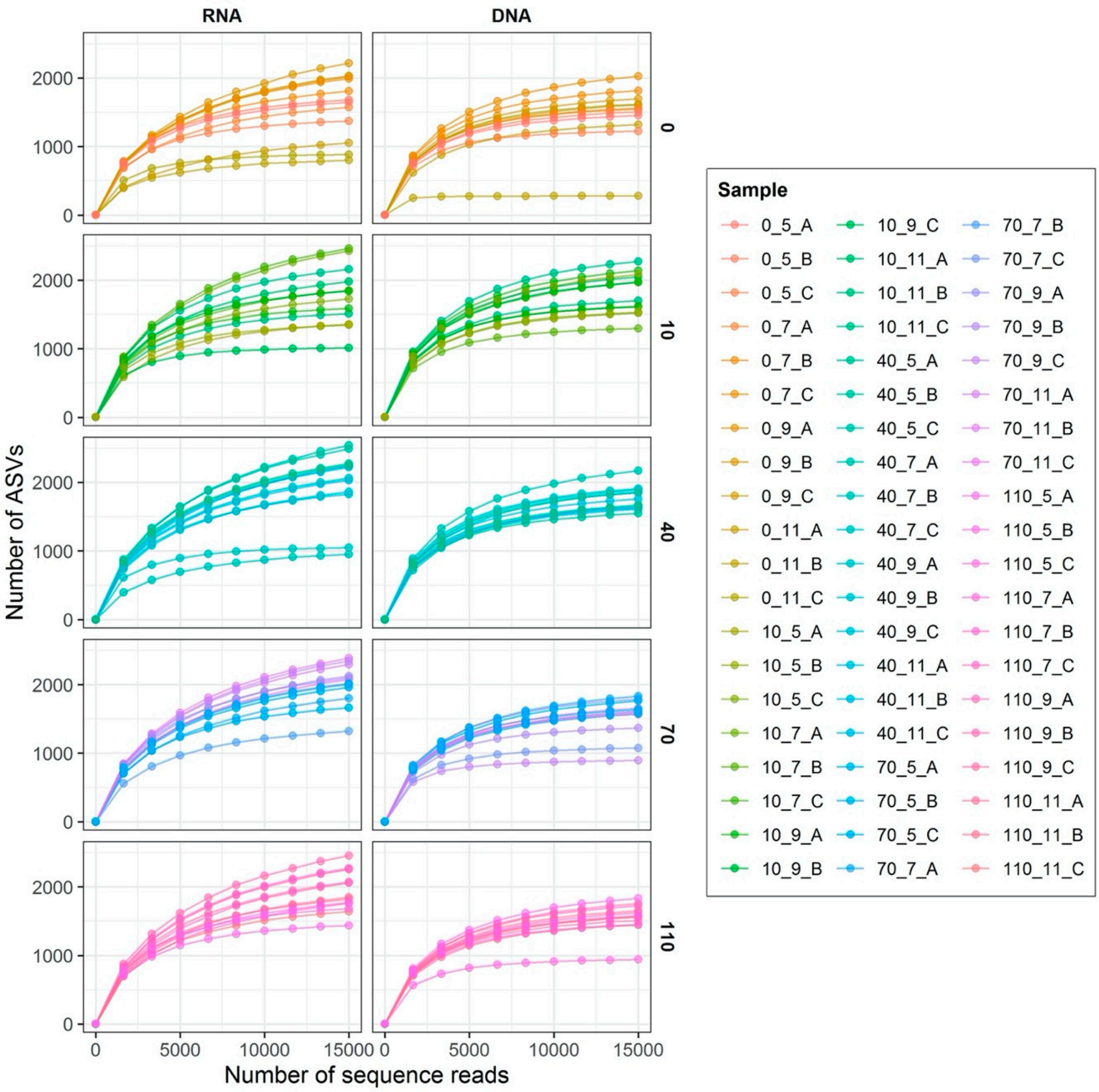

Figure S2 Line plots showing the rarefaction curves of communities profiled by (left column) RNA- and (right column) DNA-based sequencing. Colors represent samples, and distinct successional stages, sampling months, and replicates are separated by underscores in the legend panel.

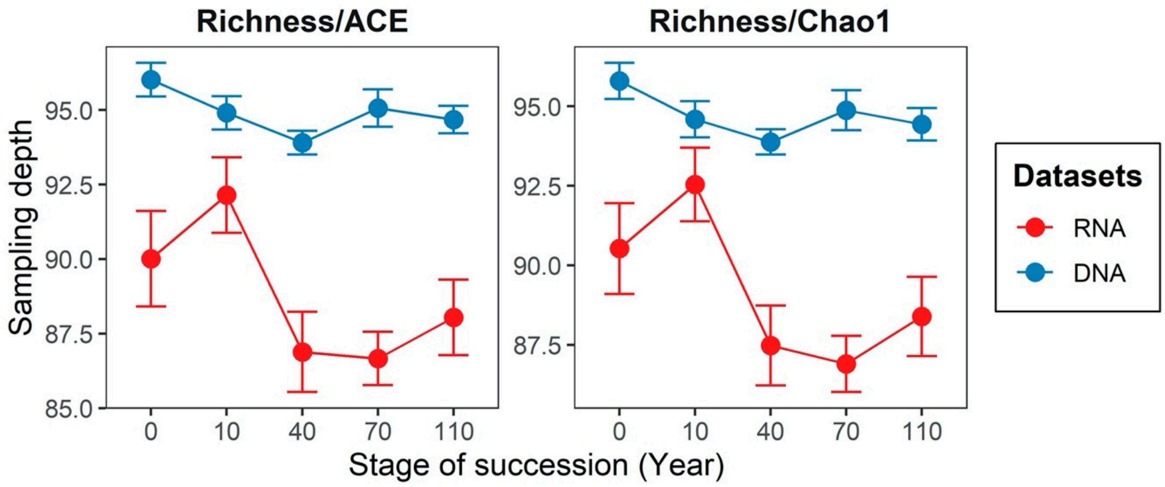

Figure S3 Sampling depths for both RNA- and DNA-based approaches. Sampling depths are indicated by the ratio between the observed richness and the estimated richness, i.e. ACE and Chao1 indexes.

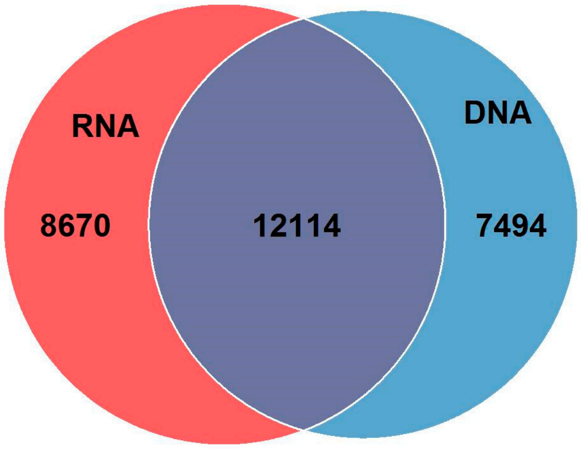

Figure S4 Venn diagram showing the overlaps of unique ASVs across RNA- and DNA-based datasets.

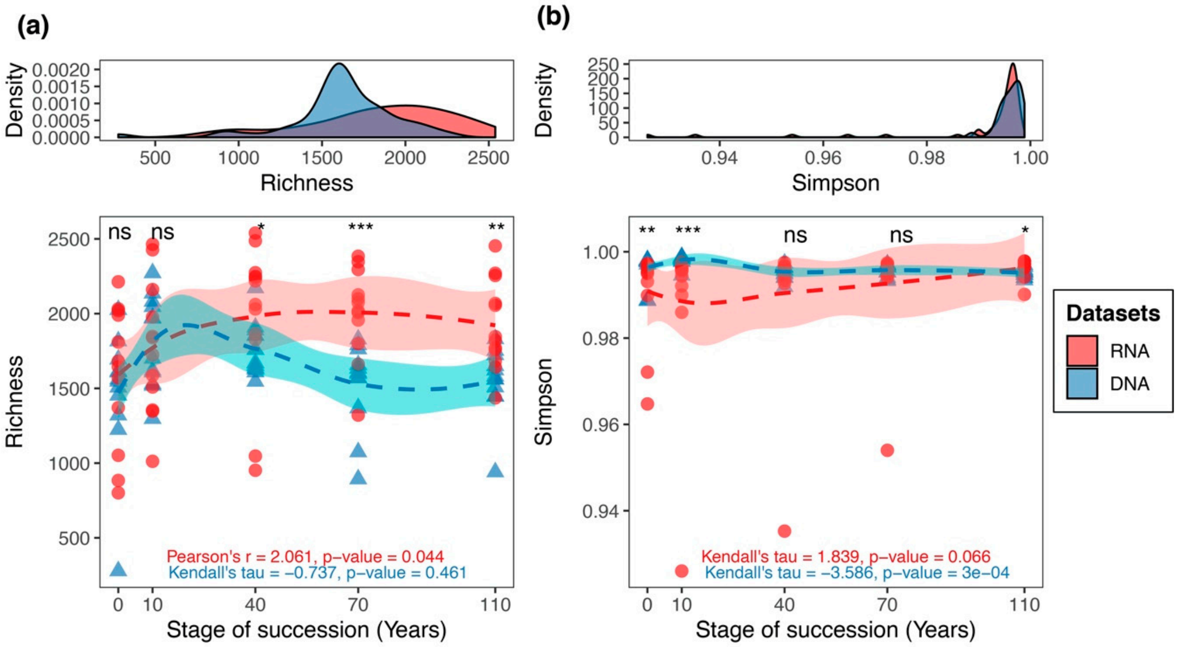

Figure S5 Changes in α-diversity along successional stages as indicated for both RNA- and DNA-based approaches; (a) observed richness and (b) Simpson index. Colors indicate the RNA- and DNA-based samples.

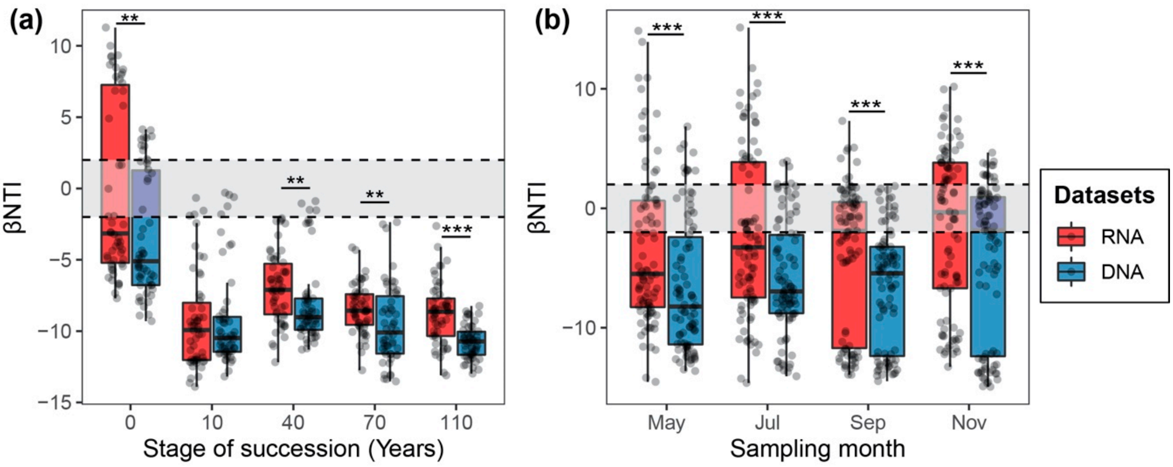

Figure S6 β-nearest taxon indexes (βNTI) between bacterial communities in (a) each successional stage and (b) each sampling month based on both RNA-based and DNA-based approaches. *** and ** indicate $P < 0.001$ and $P < 0.01$ in Wilcoxon signed-rank test, respectively.

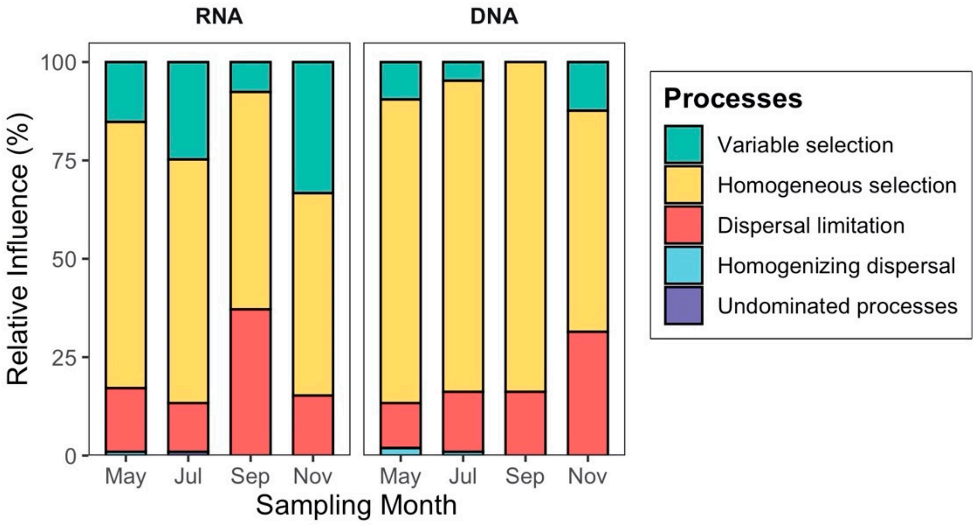

Figure S7 Stacked-bar plots showing the relative influence of distinct assembly processes structuring the spatial variation of bacterial communities in each sampling month, based on both RNA- and DNA-based approaches.

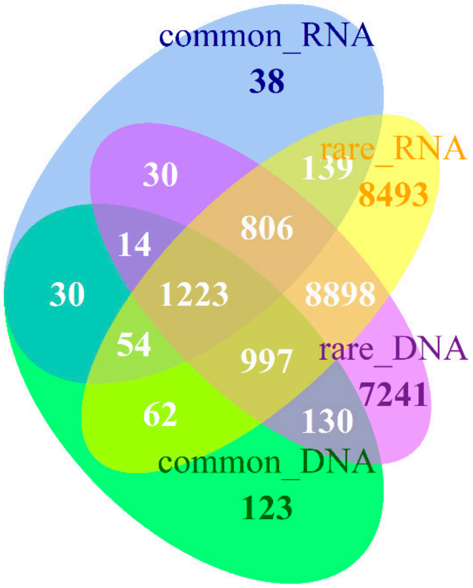

Figure S8 Venn diagram showing the overlaps of ASVs across the rare and common biospheres characterized by RNA- and DNA-based approaches. The rare biosphere was defined as a collection of taxa with relative frequencies ≤ 0.1% of the total relative abundance within a community, while the common biosphere was defined as a collection of taxa with relative frequencies > 0.1%.

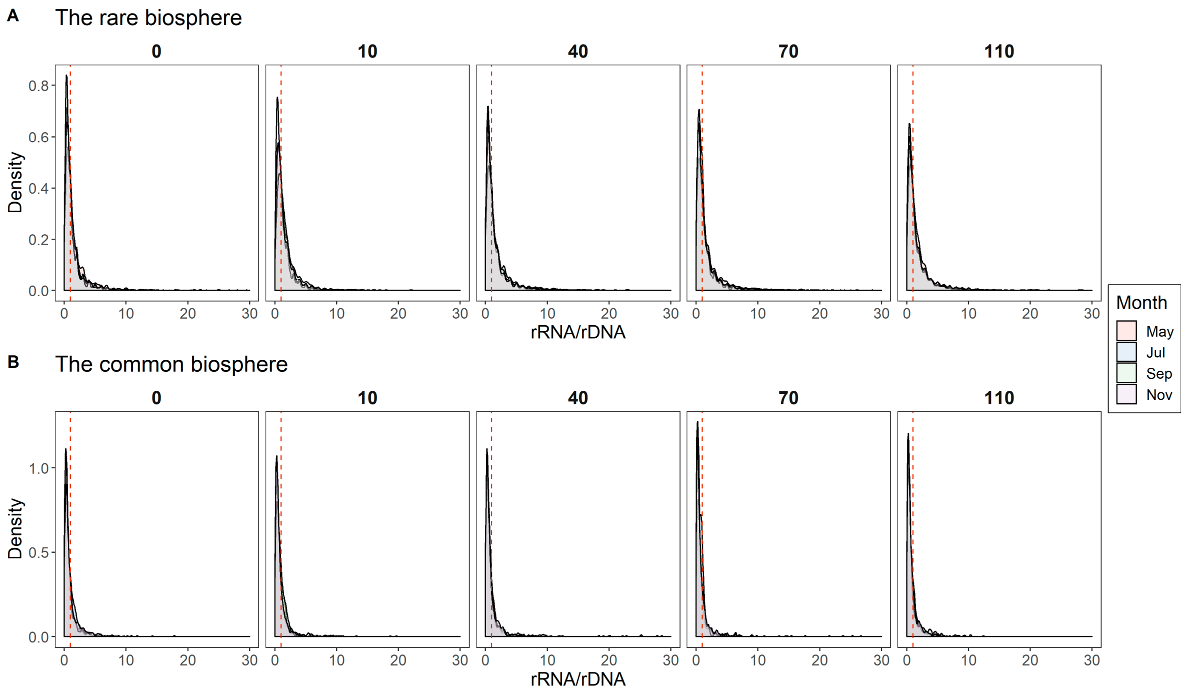

Figure S9 Density plot showing the distribution of RNA:DNA ratio in the rare and common biospheres. The rare biosphere was defined as a collection of taxa with relative frequencies ≤ 0.1% of the total relative abundance within a community, while the common biosphere was defined as a collection of taxa with relative frequencies > 0.1%.

Table S1 Coefficient of variation (cv) of α-diversity metrics (i.e. richness, Shannon index, phylogenetic diversity and Pielou's evenness) in each successional stage based on both DNA and RNA datasets. The 'Year' column indicates the successional stages (i.e., 0, 10, 40, 70 and 110 years of succession).

α-diversity indexes	Year	RNA		DNA	
		mean + se	cv	mean + se	cv
	0	1589 ± 136	29.68	1471 ± 124	29.27
	10	1771 ± 128	24.96	1813 ± 88	16.76
**Richness**	40	1982 ± 146	25.57	1765 ± 51	10.05
	70	2008 ± 87	15.05	1530 ± 82	18.62
	110	1922 ± 87	15.61	1552 ± 65	14.56
	0	6.25 ± 0.19	10.49	6.57 ± 0.15	7.85
	10	6.52 ± 0.13	6.84	6.94 ± 0.06	2.99
**Shannon**	40	6.51 ± 0.19	10.15	6.72 ± 0.05	2.55
	70	6.59 ± 0.11	6.04	6.57 ± 0.06	3.35
	110	6.67 ± 0.05	2.84	6.54 ± 0.05	2.81
	0	103.62 ± 6.67	22.29	112 ± 7.93	24.53
	10	101.1 ± 4.3	14.72	121.36 ± 4.31	12.29
**Phylogenetic diversity**	40	105.19 ± 5.23	17.23	113.22 ± 2.57	7.87
	70	105.18 ± 3.75	12.34	91.15 ± 3.15	11.99
	110	102.09 ± 2.89	9.82	97.24 ± 2.68	9.55
	0	0.851 ± 0.018	7.43	0.911 ± 0.007	2.57
	10	0.875 ± 0.013	5.34	0.927 ± 0.003	1.04
**Pielou's evenness**	40	0.861 ± 0.019	7.53	0.899 ± 0.005	1.75
	70	0.867 ± 0.011	4.2	0.899 ± 0.003	0.97
	110	0.883 ± 0.005	2.02	0.892 ± 0.002	0.77

Table S2 Three-way permutational multivariate analysis of variance (PERMANOVA) showing the influence of different factors on β-diversity of bacterial communities based on unweighted UniFrac distances. The rows 'Dataset', 'Year' and 'Month' correspond to RNA- or DNA-based datasets, successional stages, and sampling time points, respectively.

Groups	Df*	SumSqs*	MeanSqs*	F.Model*	R^2 *	*P*-values†
**Dataset**	1	1.894678	1.894678	19.50067	0.068356	**<0.001**
**Year**	4	11.41465	2.853663	29.37085	0.411816	**<0.001**
**Month**	3	0.804883	0.268294	2.761375	0.029038	**<0.001**
**Dataset:Year**	4	1.691929	0.422982	4.353476	0.061041	**<0.001**
**Dataset:Month**	3	0.352236	0.117412	1.208444	0.012708	0.1631
**Year:Month**	12	2.527413	0.210618	2.167748	0.091184	**<0.001**
**Dataset:Year:Month**	12	1.259296	0.104941	1.080091	0.045433	0.2492
**Residuals**	80	7.772774	0.09716		0.280425	
**Total**	119	27.71786			1	

* Df - degrees of freedom; SumSq - sum of squares; MeanSqs - mean of squares; F.Model - F value by permutation; R^2 - explained variation; *P*-values based on 9999 permutations. † significant *P*-values ($P < 0.001$) are shown in bold.

Table S3 Permutational multivariate analysis of variance showing the influence of successional stages (Year) and sampling time (Month) on the turnover of RNA- and DNA-based datasets (based on weighted UniFrac distances).

**Datasets**	**Groups**	**Df***	**SumSqs***	**MeanSqs***	**F.Model***	R^{2} *	***P*-values†**
**RNA**	Month	3	0.661255	0.220418	2.461365	0.051627	**0.0011**
	Year	4	6.501652	1.625413	18.15064	0.507609	**<0.001**
	Month:Year	12	2.063418	0.171951	1.920145	0.161099	**<0.001**
	Residuals	40	3.582052	0.089551		0.279665	
	Total	59	12.80838			1	
**DNA**	Month	3	0.495864	0.165288	1.577657	0.0381	0.0387
	Year	4	6.604928	1.651232	15.76083	0.507493	**<0.001**
	Month:Year	12	1.723291	0.143608	1.370719	0.13241	0.0192
	Residuals	40	4.190723	0.104768		0.321997	
	Total	59	13.01481			1	

* Df - degrees of freedom; SumSq - sum of squares; MeanSqs - mean of squares; F.Model - F value by permutation; R^2 - explained variation; *P*-values based on 9999 permutations. † significant *P*-values ($P < 0.01$) are shown in bold.

Table S4 Permutational multivariate analysis of variance showing the influence of RNA- and DNA-based dataset and sampling month on the variation of bacterial communities at each successional stage (based on weighted UniFrac distances).

Year	Groups	Df*	SumSqs*	MeanSqs*	F.Model*	R^2 *	*P*-values†
**0**	Dataset	1	0.45	0.45	3.93	0.11	**<0.001**
	Month	3	1.28	0.43	3.70	0.32	**<0.001**
	Dataset:Month	3	0.47	0.16	1.37	0.12	0.0339
	Residuals	16	1.84	0.12		0.46	
	Total	23	4.04			1	
**10**	Dataset	1	0.56	0.56	5.05	0.16	**<0.001**
	Month	3	0.75	0.25	2.28	0.22	**<0.001**
	Dataset:Month	3	0.31	0.10	0.93	0.09	0.6406
	Residuals	16	1.76	0.11		0.52	
	Total	23	3.38			1	
**40**	Dataset	1	0.94	0.94	10.10	0.30	**<0.001**
	Month	3	0.39	0.13	1.39	0.12	0.0926
	Dataset:Month	3	0.29	0.10	1.05	0.09	0.3457
	Residuals	16	1.48	0.09		0.48	
	Total	23	3.10			1	
**70**	Dataset	1	0.75	0.75	8.58	0.26	**<0.001**
	Month	3	0.44	0.15	1.68	0.15	0.0232
	Dataset:Month	3	0.28	0.09	1.06	0.10	0.3486
	Residuals	16	1.40	0.09		0.49	
	Total	23	2.87			1	
**110**	Dataset	1	0.89	0.89	11.09	0.31	**<0.001**
	Month	3	0.47	0.16	1.97	0.16	0.0083
	Dataset:Month	3	0.26	0.09	1.08	0.09	0.3144
	Residuals	16	1.29	0.08		0.44	
	Total	23	2.91			1	

* Df - degrees of freedom; SumSq - sum of squares; MeanSqs - mean of squares; F.Model - F value by permutation; R^2 - explained variation; *P*-values based on 9999 permutations. † significant *P*-values ($P < 0.001$) are shown in bold.

© 2020 by the authors. Licensee MDPI, Basel, Switzerland. This article is an open access article distributed under the terms and conditions of the Creative Commons Attribution (CC BY) license (http://creativecommons.org/licenses/by/4.0/).
